# Concept analysis of transition to motherhood: a methodological study

**DOI:** 10.4069/kjwhn.2022.01.04

**Published:** 2022-03-23

**Authors:** Woon Young Hwang, Sun Yeob Choi, Hae Jeong An

**Affiliations:** College of Nursing, Ewha Womans University, Seoul, Korea

**Keywords:** Adaptation, Concept analysis, Mothers, Transitional care

## Abstract

**Purpose:**

Although the term “transition to motherhood” is commonly used in research, the concept is not clear. This study, hence, was conducted to clarify the concept of “transition to motherhood.”

**Methods:**

The concept analysis framework developed by Walker and Avant is used to analyze the concept of transition to motherhood.

**Results:**

Transition to motherhood is defined as the physical, psychological, social, and relational (mother-baby relationship/interpersonal relationship) changes that happen to a woman after pregnancy and delivery of a baby. The attributes of the transition to motherhood include: 1) adapting to physical changes after pregnancy and childbirth; 2) experiencing various psychological changes; 3) changing of her social perception from being a woman to someone’s mother; and 4) forming and developing a relationship with the newborn, adjusting priorities, and redefining the relationship between family and others. Meeting the newborn is regarded as an antecedent of the transition to motherhood. Redefining identity and physical image, ensuring mother’s well-being, maternal attachment, and confidence in the maternal role are regarded as consequences of the transition to motherhood. The concept was clarified by the presentation of model, borderline, and contrary cases.

**Conclusion:**

The significance of this study lies in the clarification of the concept of transition to motherhood and defining its attributes. It is recommended that tools be developed to measure transition to motherhood based on the results of this study. Furthermore, nurses and midwives can use study findings to better understand the concept of transition to motherhood in providing care and support to mothers who experience it.

## Introduction

Motherhood is a state in which one experiences maternal roles [1]. It has a strong intrinsic meaning for women, such as the qualities and values of a mother, and goes beyond mere fertility [2]. Women’s motherhood transits through the stages of pregnancy, birth, and after birth [3]. During this transition, mothers report confusion due to their first experiences [4] and require a new adaptation at the physical, cultural, and social levels.

Past research has focused on the acquisition of motherhood [5,6]. Rubin [5] stated that motherhood acquisition is a part of the transition to acquiring maternal identity. Theories related to maternal identity and its acquisition were developed and tested in the 1950s and 1970s. However, these studies failed to reflect social changes such as the women’s movement and civil rights movement in the late 1960s [7]. As such, recent research deals with motherhood in a broader perspective, including women becoming mothers, bonding with the newborn, adapting to their role as a mother, and considering their roles in social contexts [8,9].

The term maternal transition is also used to mean a process that emphasizes the concept of the transition period to acquire maternity [10,11] or a goal or result to be achieved through motherhood [3,12]. The definition of the transition period to motherhood has varied from conception to postpartum, depending on how researchers have applied the concept [9,13]. Therefore, the term has been used interchangeably with antecedent factors (e.g., an encounter between a woman and child) and/or consequences (e.g., mother’s wellbeing, confidence in role performance) of the transition to motherhood, without a clear definition. Consequently, a clearer definition of transition to motherhood is required.

To date, studies related to the transition to motherhood have mainly focused on the psychological dimensions of pregnancy and giving birth [14,15]. Additionally, studies have dealt with attachment and bonding with children rather than the mother as the focal point [16,17]. In nursing studies, the transition is defined as a movement or process from one level of life to another, including changes in various dimensions such as identity, role, relationship, behavior, and ability [18]. Therefore, this study aimed to analyze and synthesize the concept of transition to motherhood across these various dimensions.

Concept analysis clarifies the meaning of an ambiguous notion and provides a clear operational definition by defining the concept and identifying its properties [19]. Walker and Avant’s [20] concept analysis method is widely used to clarify the meaning of existing concepts and develop operational definitions. As concept analysis helps to clarify nursing terms that entail unclear original meanings, it can be applied to nursing diagnosis and tool development [19]. Also, a clear concept of transition to motherhood can help nurses better understand women in the maternal transition period and enable them to provide advanced nursing care that can assist the transition process. As such, concept analysis can support providing care for women who are experiencing the transition to motherhood, conducting continuous research focusing on the concept of transition to motherhood, and facilitate effective communication between people who use the concept. Therefore, this study aimed to systematically identify the antecedent factors and consequences of the process of transition to motherhood and confirm its attributes.

## Methods

Ethics statement: This study was exempted from approval by the Institutional Review Board as it is a review of the literature using previously published studies.

### Study design

This methodological study conducted a concept analysis of the transition to motherhood by applying Walker and Avant’s [20] method through a literature review.

### Literature search and analysis

**Figure 1.**
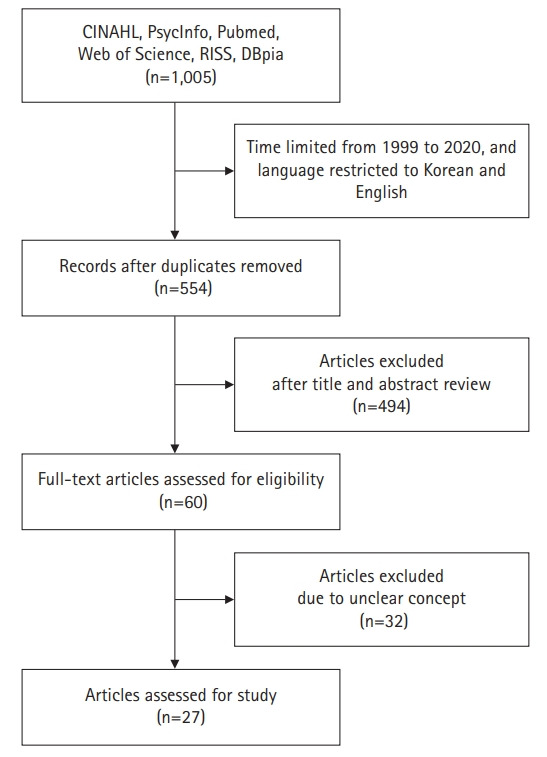
Flow diagram of literature search.

To understand how the transition to motherhood is used in literature, the following databases were searched from March 19 to April 15, 2021: the Cumulative Index to Nursing and Allied Health Literature (CINAHL), PsycINFO, PubMed, Web of Science, Research Information Sharing Service (RISS), and DataBase Periodical Information Academic (DBPia). For the search, keywords “maternal transition” and “transition to motherhood” were used. Published studies were limited to between 1999 and 2020 in the literature search, only Korean and English articles were examined, and gray literature and conference abstracts were excluded. The search initially found 1,005 titles, and 451 duplicated articles were excluded. After screening titles and abstracts, 60 articles were extracted that were considered appropriate to define the concept or explore the essential meaning of transition to motherhood. For these 60 articles, full texts were reviewed, and 27 articles met the inclusion criteria (Supplementary Table 1 [3,7-12,20-39], Figure 1).

To minimize subjectivity and ensure reliability, three researchers independently searched the literature to confirm the consistency of the search results and reached a consensus on which articles were to be included. Walker and Avant’s [20] concept analysis framework was applied to determine the scope of analysis, identify defining attributes, and propose a definition.

## Results

### Scope of use of the concept

#### Dictionary definition

There was no dictionary definition of “transition to motherhood,” so before considering the definition of ‘transition to motherhood’ in this study, the dictionary meanings of “transition to motherhood” and “transition” were examined. The Cambridge English Dictionary [40], defined “motherhood” as the fact or state of being a mother and “transition” as a change from one system or method to another, often a gradual one. Therefore, in this study, “transition to motherhood” was defined as the process of a woman becoming a mother.

#### Use of the concept in other disciplines

We reviewed the use of transition to motherhood in medicine, psychology, and women’s studies to confirm the concept in other disciplines. In the medical field, transition to motherhood was defined as a process that includes adaptation to a new stressor, physical pain, lactation, and attachment [21]. These processes were seen as dependent on the secretion of oxytocin, which starts from childbirth to the postpartum period, and plays a role in relieving maternal stress responses, supporting positive emotions, and regulating maternal behavior [21].

In psychology, the transition to motherhood involved the physical and internal changes that women acquire as mothers, which redefine their place in the sociocultural structure [8]. It is a phrase that is marked with physiological, social, and cultural changes [8]. Psychology defined the period from birth to one year as the period of maternal transition [22].

In women’s studies, transition to motherhood is defined as a dynamic, diverse, and complex process rather than a naturally ingrained, stable, and fixed process and should be interpreted within historical, cultural, social, economic, and political contexts [9]. Furthermore, transition to motherhood is a concept that includes pregnancy, childbirth, nurturing, work, leisure, and economic activity, and is defined as a period of redefining self-identity and undergoing biological changes for a woman [9,41].

#### Use of the concept in nursing

Nursing views transition to motherhood as a process of personal or interpersonal change that occurs when a woman assumes the role of a mother and evaluates herself as a mother [42]. It also considers the woman’s experiences while becoming a mother after childbirth [42] and is defined as the process of change and adaptation [23]. The term “transition to motherhood” was first used by Rubin [5] and was defined as a series of developmental processes in which a woman learns maternal roles and develops maternal identity during pregnancy and childbirth [5,43]. Mercer [44,45] further explained that becoming a mother is an intellectual process of recognizing the identity of a mother, acquiring the maternal role, and ultimately becoming a mother. In 2004, Mercer [45] defined maternity as a broad-scale transformation process from being a woman to a mother, through personal integration by restructuring reality, rather than simply acquiring it, and recommended that the term maternal acquisition be withdrawn. Roy [46] interpreted maternal transformation as an adaptation based on adaptation theory and viewed it as a process of adapting to changes that come with the commencement of the mother’s role, along with changes in relationships, ability, and behaviors after childbirth [24]. It is interpreted as a concept that includes physical and psychological changes from pregnancy to postpartum [25,47]. Moreover, the transition to motherhood is viewed as a period in which women change their perception of themselves [26,48] and interpret the transition as a process of forming a new identity [26]. Therefore, change to maternal identity in nursing can be defined as a process of adaptation to becoming a mother, as a woman plays the role of a mother and establishes an identity during her pregnancy and the postpartum period.

Considering the concept of transition to motherhood as described above, physical, sociocultural, and economic dimensions of maternal transformation were identified, while in nursing studies, it appeared to mainly focus on the adaptation to motherhood. However, considering that women’s social activities are increasing socioculturally and that the transformation process includes changes in multiple dimensions such as identity, roles, and relationships [18], it is necessary to define and clarify the concept of transition to motherhood in a wider range of dimensions, including social and relational aspects.

### Defining attributes and proposed definition

#### Defining attributes of transition to motherhood

Attributes are the key characteristics that simultaneously appear with the concept [49]. From the literature surveyed for this study, the concept of transition to motherhood was defined or used as follows:

① A significant event in a woman’s life, that requires adjustments on physical, biological, psychological, cultural, and social levels [49]

② A process of response and adaptation that changes according to an individual’s continuous assessment of a situation [23]

③ Transformation process of the self through complex physical, cognitive, behavioral, and emotional changes [26]

④ A woman’s reactions to the concrete physical changes [27]

⑤ A changing conception of self as related to others [28]

⑥ Integrating the role of a mother into her sense of self and forming a relationship with her infant [23]

⑦ A dialog between internal and external positions, where internal positions are perceived as part of oneself and external as part of the environment [8]

⑧ Forging a new identity by a woman as a mother, that revolves around her functioning in a new role [29]

⑨ Process of personal or interpersonal change that occurs by taking on the role of a mother and evaluating oneself as a mother [23]

⑩ A stressful experience, generating a sense of loss in terms of autonomy, time, appearance, and occupational identity [30]

### Proposed definition of transition to motherhood

Based on these attributes, transition to motherhood is a continuous process of adapting to and responding to constantly changing physical, psychological, social, and relational dimensions, from being a woman to becoming someone’s mother (Table 1).

### Case examples: model case

The following model case illustrates the concept of transition to motherhood, while demonstrating all the defining attributes [20].

‘M’ is a 35-year-old woman who became pregnant 3 years after marriage. M’s weight increased by 20 kg, and she had morning sickness throughout her pregnancy. A week had passed since childbirth, but she did not lose weight as quickly as expected. Every 3 to 4 hours, she experienced breast engorgement with breastmilk leaking. Although there were difficulties with these physical changes, she expected all these as she had heard about them from the nurse at discharge education. She adapted physically without much difficulty thanks to the advice from the nurse and help from online community peers who experienced childbirth recently (physical dimension). The posture of holding and breastfeeding a newborn was initially uncomfortable, and even when changing diapers or bathing the baby, she was cautious and clumsy in handling the baby. She felt incompetent and there was nothing she could do. However, as the days went by, she gradually got used to it and gained confidence; she felt happy to see her baby growing daily (psychological dimension). In comparison to being called by her own name as before, after birth she is now called “Amy’s mom”; it was unfamiliar, and she felt like she had lost herself (social dimension). However, seeing her newborn completely dependent on her, she felt that her existence was necessary and felt proud and infinitely happy to be a mother (mother-newborn relational dimension). M and her husband loved each other passionately and put each other first. However, since the birth of their baby, they were no longer the priority for each other. Initially, both the husband and M felt awkward and some sadness, but as time progressed, they felt a sense of happiness in their new family relationship (interpersonal relationship).

As seen in this transition to motherhood, M faced difficulties in the early stages of the transition to motherhood but gradually adapted to and overcame them.

### Developing additional examples

#### Borderline case

As a borderline case includinig some important attributes of the concept [20], the following is presented:

‘B’ is a 17-year-old high school student who became pregnant and dropped out of school. B gave birth by cesarean section 4 days ago and is currently recovering. After giving birth, B became aware of the physical changes, such as secretion of breastmilk and surgical scarring in her body that was brought on by motherhood. Upon seeing the bloody lochia, B felt upset as she thought that she was sick (physical dimension). B was afraid of these changes in her body, so she asked the nurse and was relieved to hear the nurse explain that these changes were a normal process. B also felt joy in the fact that the painful process had ended and that she could return to the life before pregnancy. However, she felt sad that she now has a surgical scar on her body (emotional dimension). At the time of breastfeeding, B felt confused when the nurse called her “Jane’s mother” or “mommy” and did not call her by her own name (social dimension). Additionally, during breastfeeding time, all the attention and affection of her boyfriend and her parents were toward the newborn. B also felt that the nurse was only interested in the baby’s condition during breastfeeding and was indifferent to her feelings and changes, which made B feel alienated and jealous of her baby. Thus, she felt that she is no longer a priority and felt irritable returning to life as before no longer was possible.

This case contains the physical, psychological, and social attributes of the transition to motherhood, where B was aware of her physical and psychological changes (physical and psychological dimension) and also was recognized and referred to as a mother by those around her (social dimension). However, she failed to form and develop a relationship with her newborn and had not reconstructed her relationship with her boyfriend as parents. Thus, this is an example in which the properties of the relational dimension of transition to motherhood are excluded.

#### Contrary case

A contrary case is a clear example of something that is not a defined concept, which does not contain any of the important properties of the said concept [20], as illustrated in the following case:

‘C’ is a 32-year-old woman who has been married for 5 years, as well as a working woman who exercises every day, takes care of herself thoroughly, and is recognized at work. Over the past year, she had been desperately wanting a baby and had undergone artificial insemination and in vitro fertilization several times but failed. After long consideration, C adopted a 3-month-old girl and decided to raise her well. During the daytime a babysitter cared for the baby, and C spent time with the baby after work. However, C became increasingly tired and overwhelmed as she watched the baby cry constantly. Despite trying to figure out why the baby was crying, her efforts were in vain and she grew to resent the baby. She told her husband that raising a child was not easy and that she had found it difficult, but instead of consoling and offering support, he said that it was her choice and that she should do her best in the role. C thought that she would be happy when she had a baby, which she so desperately wanted; However, she increasingly disliked the baby and thought it was because the child was not hers biologically. Her sense of burden in caring for the baby became heavier over time, in contrast to the respect she enjoyed at work. Therefore, she worked hard to build her career without caring for her daughter after work hours. One day, the child continued crying for no apparent reason and she took her to the emergency room. The nurse asked her about the baby, but she could not answer the question properly because she could not figure out the meaning of her daughter’s usual crying. She felt so exhausted and miserable at the thought that she could do nothing as a mother, and grabbing the nurse, cried.

In this case, the attributes of physical, psychological, social, and relational dimensions are not included in C’s transition to motherhood.

### Antecedents and consequences of the transition to motherhood

**Figure 2.**
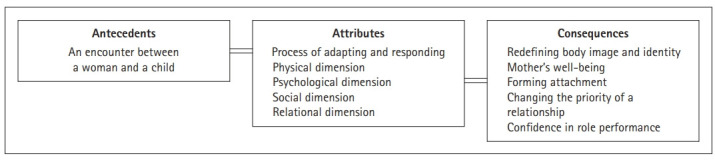
Conceptual structure of transition to motherhood.

Antecedents refer to events or circumstances that must occur before the transition to motherhood, and outcome factors refer to consequences that occur because of the transition to motherhood [20] (Figure 2).

### Antecedents

As defined in this study, an encounter between a woman and a baby must precede the transition to motherhood. Through pregnancy and childbirth, a woman recognizes her baby by interacting with the fetal movements, and through morning sickness and delivery process. The mother goes through a process of adaptation at the physical, psychological, social, and relational levels. This is similar to previous studies which emphasized that the birth of a baby is essential for maternal transformation [8] and that transition to motherhood proceeds through pregnancy and childbirth [25,43]. Furthermore, even in an adoption situation, maternal change can occur as women adapt to maternal role and overcome difficulties [41]. To bond with the newborn during the transition to motherhood, women need coping strategies, caring skills [27], and support from family members and partners [25].

### Consequences

As a result of the transition to motherhood defined in this study, women integrate and balance their roles as an independent person and as a mother. Consequently, the woman forms a relationship with her baby, takes on the maternal role, reshapes her identity, and generally adapts soon after pregnancy and childbirth. Additionally, by redefining relationships with others and adjusting priorities, she adapts and grows while balancing social and maternal roles. As women undergo maternal changes, it eventually leads to a new definition of body image and identity [8].

Women who experience difficulties in this process face challenges recognizing themselves as a mother, thus perceiving their baby negatively [23]. Consequently, the newborn’s growth and development are negatively impacted, and they have difficulties in forming attachments [50]. It can cause women to experience negative emotions such as depression, helplessness, and guilt about not being able to master the role of a mother properly [27].

Alternatively, however, if the transition to motherhood is successful, women gain confidence in performing the mother’s role, thus improving their sense of efficacy as a mother [27]. This helps form bonding with and attachment to the baby [8,9] and positively affects physical, socioemotional, language, and cognitive development [51,52]. Additionally, through a successful transition to motherhood, women can develop their maternal behavior and continue to grow [51], which ultimately has a positive effect on the well-being and mental health of mothers, and subsequently, the newborn [8,9].

### Empirical indicator

An empirical indicator refers to an observable index that defines the properties of a concept [20]. For the psychological dimension, the Differential Emotions Scale [53] was used in a study by Behringer et al. [54] to measure women’s emotions over time. The scale includes 10 basic emotions during pregnancy or childbirth, such as joy, sadness, fear, and anger. Mortazavi et al. [55] used the General Health Questionnaire-28 (GHQ-28) [56] to investigate the psychological status of women in the 3rd trimester of pregnancy and 8 weeks after childbirth. The subitems comprise four themes: (1) physiological symptoms, (2) anxiety and insomnia, (3) social dysfunction, and (4) severe depression. This tool measures negative psychological states and is widely used to measure the psychological dimension involved in the transition to motherhood [52,57]. However, it is limited in measuring the overall psychological changes of women during pregnancy or after childbirth, including positive psychological states. An additional limitation of GHQ-28 is that although it includes the psychological state in the physical and social dimensions, it does not include relational dimensions. In contrast, for the relational dimension, the Prenatal Attachment Inventory [58] has been used to measure mother-baby bonding. This tool is widely used to measure the relational aspects of mother and baby, and is evaluated to have high validity [59]. However, it was difficult to find a tool to measure the interpersonal aspect of the relational dimension.

As seen through the above tools, only some attributes of transition to motherhood have been measured in previous studies [55,60]. To confirm the empirical indicator for transition to motherhood, it is necessary to develop a tool that can comprehensively evaluate all the dimensions of attributes of the concept. Based on synthesis of the concepts used in previous studies to measure the transition to motherhood, the following empirical criteria should be considered: body image, psychological response related to pregnancy and childbirth, quality of life, maternal identity, self-awareness, ego-identity, attachment, bonding, maternal efficacy, parenting confidence, parenting burden, and spousal support.

## Discussion

This study aimed to systematically identify the antecedent factors and consequences of the concept, transition to motherhood.

Among the attributes of transition to motherhood confirmed in this study, the psychological dimension includes simultaneously feeling different emotions during the transition to motherhood. In previous studies, when measuring the psychological dimension, negative emotions such as depression [29] and worry [32] were mainly addressed. However, it is necessary to consider that various positive and negative emotions can coexist in the psychological dimension of the transition to motherhood.

In some studies, the period and timing of maternal transition were mostly defined as the period from pregnancy to the first year after childbirth [9,13]. The transition period is not defined as a continuous occurrence throughout life [8,23]. Therefore, the period of transition to motherhood confirmed in this study is a period that occurs intensively in the early stages after childbirth. Subsequently, it can be regarded as a process of continuous adaptation and reaction as the newborn grows.

The antecedent for the transition to motherhood derived from this study was the encounter between the mother and the baby. Most studies on the transition to motherhood have been conducted on mothers who have undergone pregnancy and childbirth [9,49]. However, given that social change influenced by modernization has influenced maternity to be viewed as a sociocultural composition, rather than a biological one, it is necessary to include adoption in the discussion of maternal transformation [31,61]. Thus, it is important to understand that the encounter with a baby as a leading factor in this study occurs in the relational exchange between the mother and baby, rather than focusing only on biological pregnancy and childbirth.

The consequences derived from this study were a redefinition of body image and identity, mother’s well-being, formation of attachment, change of priorities in relationships, and confidence in role performance. This implies that motherhood is not limited to a specific dimension, such as the physical or psychological dimension, but is a multidimensional total adaptation. Unlike previous studies that focused on the baby, such as attachment felt towards the baby, this result draws attention to the multidimensional characteristics of women’s adaption and their sense of well-being as a mother. Through this, the woman integrates and balances herself, both as an individual and as a mother.

In conclusion, the concept of transition to motherhood in previous studies was focused on the mother’s role in delivering and caring for her infant. Moreover, the focus was on the infant, such as attachment to and the skills required to care for the infant. In this study, however, four attribute dimensions were identified: 1) the physical dimension, which is the process of adaptation to the changing body after pregnancy and childbirth; 2) the psychological dimension, which is the process of experiencing various positive and negative psychological changes, such as joy, fulfillment, anxiety, helplessness, and loneliness; 3) the social dimension, which is the process of changing the social perception from being a woman to being someone’s mother; and 4) the relational dimension, which is the process of forming and developing relationships with the baby, and redefining relationships with family and others.

This study confirmed that in addition to being a mother, the woman experiences and adapts to multidimensional changes during pregnancy and after childbirth. Therefore, nurses and midwives can build on understanding the changes in physical, psychological, social, and relational aspects experienced by women in the maternal transition period and provide nursing care according to these various dimensions. Consulting and continuously providing advice on the various aspects of transition that women experience after childbirth may promote women’s successful transition to motherhood. This study is significant as it provides basic data for providing holistic nursing by clearly establishing the concept of transition to motherhood. Given that this is limited to review of the literature, future studies that explore and determine whether the attributes of the transition to motherhood identified in this study reflect women’s actual experiences, will be beneficial. Moreover, by reflecting on these identified attributes and empirical indicators of maternal transformation, we propose a future study to develop a tool for measuring the transition to motherhood and nursing interventions to help mothers successfully transition to motherhood.

## Figures and Tables

**Table 1.** Proposed definition of transition to motherhood

Dimension		Attributes
Physical		The process of adapting to the changing body after pregnancy and childbirth.
Psychological		The process of experiencing various positive and negative psychological changes such as joy, fullness, anxiety, incompetence, loneliness.
Social		The process of social perception changes from one woman to someone’s mother.
Relational	Mother-baby	The process of building and developing a relationship with a child.
	Interpersonal	The process of redefining relationships with family and others including the priorities.

## Supplementary materials

Further details on supplementary materials are presented online (available at https://doi.org/10.4069/kjwhn.2022.01.04).

Supplementary Table 1. List of the Included Studies
