# Supplementary material for: Concept analysis of transition to motherhood: a methodological study
Source: Korean J Women Health Nurs. 2022 Mar 23;28(1):8–17. doi: 10.4069/kjwhn.2022.01.04 (PMC9334210; doi:10.4069/kjwhn.2022.01.04)
Supplement: Supplementary Table 1. — List of the Included Studies [file kjwhn-2022-01-04suppl.pdf]

Supplementary Table 1. List of the Included Studies

Reference No.	Included Studies
[25]	Aber C, Weiss M, Fawcett J. Contemporary women's adaptation to motherhood: the first 3 to 6 weeks postpartum. Nurs Sci Q. 2013;26(4):344-351. https://doi.org/10.1177/0894318413500345
[21]	Bell AF, Erickson EN, Carter CS. Beyond labor: the role of natural and synthetic oxytocin in the transition to motherhood. J Midwifery Womens Health. 2014;59(1):35-108. https://doi.org/10.1111/jmwh.12101
[22]	Camberis AL, McMahon CA, Gibson FL, Boivin J. Age, psychological maturity, and the transition to motherhood among English-speaking Australian women in a metropolitan area. Dev Psychol. 2014;50(8):2154-2164. https://doi.org/10.1037/a0037301
[36]	Churchill AC, Davis CG. Realistic orientation and the transition to motherhood. J Soc Clin Psychol. 2010;29(1):39-67. https://doi.org/10.1521/jscp.2010.29.1.39
[35]	Darvill R, Skirton H, & Farrand P. Psychological factors that impact on women's experiences of first-time motherhood: a qualitative study of the transition. Midwifery. 2010;26(3):357-366. https://doi.org/10.1016/j.midw.2008.07.006
[24]	Erfina E, Widyawati W, McKenna L, Reisenhofer S, Ismail D. Exploring Indonesian adolescent women's healthcare needs as they transition to motherhood: a qualitative study. Women Birth. 2019;32(6):e544-e551. https://doi.org/10.1016/j.wombi.2019.02.007
[31]	Fontenot HB. Transition and adaptation to adoptive motherhood. J Obstet Gynecol Neonatal Nurs. 2007;36(2):175-182. https://doi.org/10.1111/j.1552-6909.2007.00134.x
[7]	Fouquier KF. The concept of motherhood among three generations of African American women. J Nurs Scholarsh. 2011;43(2):145-153. https://doi.org/10.1111/j.1547-5069.2011.01394.x
[34]	Holden L, Hockey R, Ware R, Lee C. Mental health-related quality of life and the timing of motherhood: a 16-year longitudinal study of a national cohort of young Australian women. Qual Life Res. 2018;27(4);923-935. http://doi.org/10.1007/s11136-018-1786-7
[26]	Lawler D, Begley C, Lalor J. (Re)constructing myself: the process of transition to motherhood for women with a disability. J Adv Nurs. 2015;71(7):1672-1683. https://doi.org/10.1111/jan.12635
[39]	Lee SC, Keith PM. The transition to motherhood of Korean women. J Comp Fam Stud. 1999;30(3):453-470. DOI: 10.3138/jcfs.30.3.453
[38]	Millward LJ. The transition to motherhood in an organizational context: An interpretative phenomenological analysis. J Occup Organ Psych. 2006;79(3):315-333. https://doi.org/10.1348/096317906X110322
[33]	Missal B. Gulf Arab women's transition to motherhood. J Cult Divers. 2013;20(4):170-176.Churchill AC, & Davis CG. Realistic orientation and the transition to motherhood. J Soc Clin Psychol. 2010;29(1):39-67. https://doi.org/10.1521/jscp.2010.29.1.39
[37]	Nelson AM. Transition to motherhood. JOGNN. 2003;32(4): 465-477. https://doi.org/10.1177/0884217503255199
[23]	Shin HJ. Maternal transition in mothers with high risk newborns. J Korean Acad Nurs. 2004;34(2):243-251. https://doi.org/10.4040/jkan.2004.34.2.243
[28]	Smith JA. Identity development during the transition to motherhood: an interpretative phenomenological analysis. J Reprod Infant Psycol. 1999;17(3):281-299. https://doi.org/10.1080/02646839908404595
[10]	Spector MG, Cinamon RG. Identity exploration during the transition to motherhood: facilitating factors and outcomes. Career Dev. Int. 2017;22(7):829-843. https://doi.org/10.1108/CDI-01-2017-0021
[32]	Spinelli M, Frigerio A, Montali L, Fasolo M, Spada MS, Mangili G. 'I still have difficulties feeling like a mother': The transition to motherhood of preterm infants mothers. Psychol Health. 2016;31(2):184-204. https://doi.org/10.1080/08870446.2015.1088015
[29]	Talmon A, Horovitz M, Shabat N, Haramati OS, Ginzburg K. "Neglected moms" - the implications of emotional neglect in childhood for the transition to motherhood. Child Abuse Negl. 2019;88:445-454. https://doi.org/10.1016/j.chiabu.2018.12.021
[27]	Talmon A, Shaham Salomon N, Ginzburg K. Differentiation of the self and the body and adjustment to motherhood: a latent class analysis. J Affect Disord. 2020;276:287-296. https://doi.org/10.1016/j.jad.2020.07.093
[30]	Taubman-Ben-Ari O, Shlomo SB, Sivan E, Dolizki M. The transition to motherhood: a time for growth. J Soc Clin Psychol. 2009;28(8):943-970. https://doi.org/10.1521/jscp.2009.28.8.943
[12]	Tekavc J, Wylleman P, Cecić Erpič S. Becoming a mother-athlete: female athletes' transition to motherhood in Slovenia. Sport Soc. 2020;23(4):734-750. https://doi.org/10.1080/17430437.2020.1720200
[8]	Uriko K. Dialogical self and the changing body during the transition to motherhood. J Constr Psychol. 2019;32(3):221-235. https://doi.org/10.1080/10720537.2018.1472048
[3]	van Vugt E, Versteegh P. "She gave me hope and lightened my heart": The transition to motherhood among vulnerable (young) mothers. Child Youth Serv Rev. 2020;118:105318. https://doi.org/10.1016/j.childyouth.2020.105318.
[20]	Walker LO, Avant KC. Strategies for theory construction in nursing. 6th ed. New York, NY: Pearson/Prentice Hall Upper Saddle River; 2019. 251 p.
[11]	Wu WR, Hung CH. First-time mothers psychiatric health status during the transition to motherhood. Community Ment Health J. 2016;52(8):937-943. https://doi.org/10.1007/s10597-015-9892-2
[9]	Yopo Díaz M. Enacting motherhood: time and social change in Chile. J Gend Stud. 2018;27(4):411-427. https://doi.org/10.1080/09589236.2016.1223619

https://doi.org/10.4069/kjwhn.2022.01.04
